# Edge CA-CFAR Data Reduction for Bandwidth-Efficient Real-Time Wideband Spectrum Sensing on Low-Cost SDRs

**DOI:** 10.3390/s26144468

**Published:** 2026-07-14

**Authors:** Yunsu Bae, Hajung Lee, Hyojun Park, Won-ho Jang, Byung-Jun Jang

**Affiliations:** 1 Department of Electrical Engineering, Kookmin University, Seoul 02707, Republic of Korea; bys705@kookmin.ac.kr (Y.B.); chris020617@kookmin.ac.kr (H.L.); joon_1234@kookmin.ac.kr (H.P.); 2 SUNI Corporation, #516, #517, Incubation Center, 55, Hanyangdaehak-ro, Ansan 15588, Gyeonggi-do, Republic of Korea; whj@suni-wpt.ai

**Keywords:** spectrum sensing, software-defined radio, CA-CFAR, FPGA, GPU, wideband monitoring, real-time processing, edge computing

## Abstract

Real-time wideband radio frequency (RF) spectrum monitoring is increasingly important for unmanned aerial vehicle (UAV) detection and RF surveillance. Low-cost software-defined radio (SDR) networks are attractive but constrained by limited instantaneous bandwidth per node, I/Q data transfer bottlenecks over USB 2.0, and multi-node computational overhead. This paper proposes a bandwidth-efficient FPGA-GPU heterogeneous architecture addressing these limitations. A hardware-efficient cell-averaging constant false alarm rate (CA-CFAR) IP core is deployed on the edge FPGA of each SDR node, forwarding only signal-containing intervals to reduce data transfer volume proportionally to the target duty cycle. Spectra from multiple nodes are stitched into a wideband view and processed in real time via a GPU-accelerated pipeline. The CA-CFAR IP occupies 16.3% of available LUTs with no BRAM and a fixed 10-cycle latency at 100 MHz. Experiments on a five-SDR testbed demonstrate an 88% data transfer reduction at a 10% duty cycle, 376 μs latency from signal acquisition to display-buffer preparation, 96.26% detection probability at −83.16 dBm (SNR ≈ 13 dB), and a 4.5× to 6.0× GPU speedup over CPU processing. These results support real-time wideband RF monitoring on resource-constrained SDR platforms.

## 1. Introduction

The rapid proliferation of wireless technologies in autonomous driving, aviation, defense and communications has led to a substantial increase in the use of the radio frequency spectrum. At the same time, research on spectrum sensing, which enables real-time monitoring and identification of diverse radio frequency (RF) signals in complex electromagnetic environments, has accelerated significantly [1,2,3]. This real-time RF detection capability has become a core component of modern surveillance architectures, ranging from civilian security systems to military electronic warfare platforms [4]. In particular, unmanned aerial vehicles (UAVs) targeting urban areas and major national security facilities have recently emerged as a significant threat. Consequently, wideband spectrum coverage has become important for monitoring noncooperative targets that may employ sophisticated evasion strategies [5,6,7,8].

Noncooperative targets often conceal their emissions by using frequency hopping and short-pulse waveforms to avoid detection. In a congested spectrum containing mixed wireless communication and radar emissions, detecting signal-present intervals in real time directly determines the reliability of an RF surveillance system. However, single-node surveillance systems suffer from two fundamental limitations: non-line-of-sight (NLoS) obstruction caused by surrounding terrain and the physical constraint on instantaneous bandwidth. To overcome these issues, distributed sensor networks that deploy multiple sensors across different spatial locations have emerged as an effective alternative [9,10]. As a result, modern RF surveillance architectures must jointly satisfy wideband coverage, real-time detection performance and cost effectiveness, given the need to operate multiple sensor nodes.

Conventional systems have typically relied on expensive RF front ends with wide instantaneous bandwidth and dedicated high-speed signal processors [11,12]. However, this hardware-centric approach entails substantial deployment cost, which limits scalability in distributed surveillance networks. To mitigate these drawbacks, low-cost software-defined radio (SDR)-based systems have been actively investigated [13,14,15]. Nevertheless, SDR-based platforms are constrained not only by ADC/DAC bandwidth but also by the transfer rate limits of host interfaces such as USB 2.0 and Ethernet when large volumes of raw I/Q data are processed [16,17]. When multiple sensor nodes operate simultaneously, the data generated by each node can reach tens to hundreds of megabytes per second, and transmitting such data in raw form to a central host causes network saturation and transfer delays. These bandwidth constraints fundamentally limit real-time operation regardless of processing capability.

As summarized in Table 1, recent RF-based UAV detection studies have improved signal identification using SDR measurements and deep-learning classifiers [7,8,13], while SDR/RFNoC-based monitoring systems and SDR testbed studies have shown wideband or measurement-based spectrum observation in practical RF environments [2,14]. FPGA-based CFAR implementations and GPU radar-processing studies have also reduced detection or processing latency at individual processing stages [18,19,20,21].

**Figure 1:**
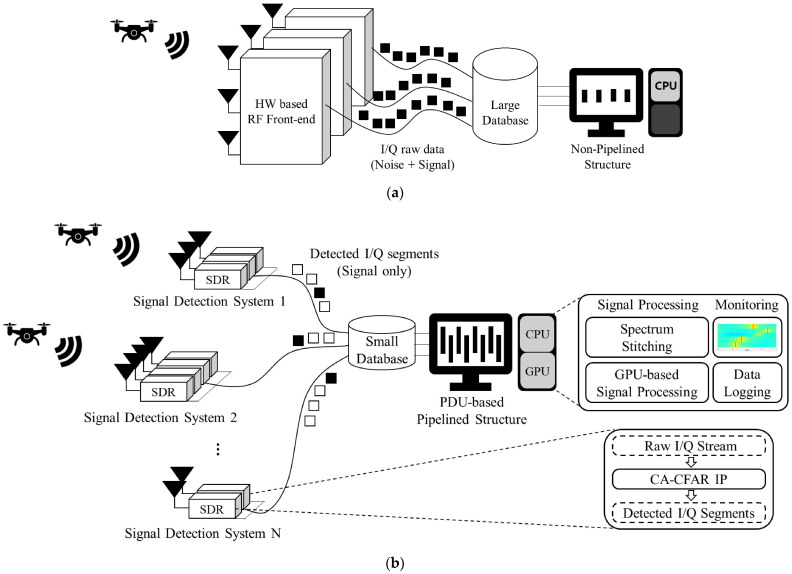
Comparison of spectrum sensing architectures: (**a**) conventional high-cost hardware-centric system suffering from data bottlenecks; (**b**) proposed FPGA-GPU acceleration system employing edge-based data reduction.

Accordingly, this paper proposes a real-time wideband RF spectrum sensing system that combines edge-side data reduction with host-side GPU processing to address both the data transfer overhead and the computational bottleneck. As illustrated in Figure 1b, the proposed system performs cell-averaging constant false alarm rate (CA-CFAR)-based preprocessing on the FPGA connected to each SDR node to suppress noise-only samples before transmission and then uses GPU parallelism to process spectra from multiple channels. This hybrid approach is intended to reduce both host-interface traffic and host-side computational load. The main contributions of this work are as follows:

- Edge-side data reduction via a hardware-efficient CA-CFAR IP core is designed for the Zynq-7010 FPGA in each SDR node: It uses in-place recursive summation and arithmetic right-shift division to achieve O(1) per-sample computational complexity for the reference-cell summation update, independent of the number of reference cells, by using only the newly entering and departing samples instead of recomputing the full window at each clock cycle. The design occupies 16.3% of the available lookup tables (LUTs) and consumes no block RAM (BRAM) resources while enabling real-time operation with a fixed latency of 10 clock cycles at 100 MHz. Using this edge-based data reduction technique, 88% of host-bound data is removed in a 10% duty-cycle environment, alleviating the transfer throughput limitation.
- PDU-based multi-SDR event aggregation and parallel multi-SDR control: To compensate for the narrow instantaneous bandwidth of a single low-cost SDR, a multithreaded software architecture is implemented for stable parallel operation of multiple SDR nodes. Because the SDR nodes cover non-overlapping sub-bands, the host pipeline uses protocol data unit (PDU) metadata such as SDR identifier, center frequency, bandwidth and timestamp to preserve source identity and aggregate detected events into a stitched wideband view. The PDU-based scheduling pipeline also provides a structured input unit for GPU-accelerated computation, enabling low-latency signal processing.

To validate the proposed system, a custom hardware testbed was constructed and evaluated at the system level. Experimental results demonstrate that the system achieves up to 88% reduction in data transfer volume relative to conventional approaches, a latency of 376 μs from signal acquisition to display-buffer preparation, a detection probability exceeding 95% ($P_{d}$ = 96.26%) at −83.16 dBm received power (noise floor −96 dBm) and a 4.5× to 6.0× processing speed-up over the tested CPU-based computation. These results demonstrate that the proposed architecture can support real-time wideband RF monitoring on resource-constrained SDR platforms.

The remainder of this paper is organized as follows. Section 2 presents the proposed system architecture from two complementary perspectives. Section 3 describes the hardware and software implementation of each component. Section 4 reports the experimental setup and quantitative performance evaluation. Section 5 concludes the paper and outlines future research directions.

## 2. Proposed System Architecture

The proposed RF spectrum sensing system consists of two principal processing stages: (1) an RF front end and preprocessing stage that collects signals from multiple SDR nodes and performs FPGA-based data reduction and (2) a host-side acceleration stage responsible for data aggregation, pipeline control and high-speed GPU parallel computation.

### 2.1. FPGA-Based CA-CFAR Design for SDR Nodes

In electronic warfare (EW) and signal intelligence (SIGINT) environments, the parameters of RF signals emitted by a target are unknown a priori. Accordingly, the spectrum detector must identify unknown signals without prior knowledge of waveform, modulation scheme or bandwidth. Because conventional matched filter-based pulse compression requires a replica of the transmitted signal, it cannot be applied in this scenario [27]. Therefore, this work adopts instantaneous power-based detection of received I/Q data to determine whether a signal is present [28].

In practice, RF signals emitted by a target are received by the SDR antenna and RF transceiver, converted into digital samples by the ADC and forwarded to the FPGA. Since the proposed system operates multiple SDR nodes in parallel for wide-area and wideband coverage, the resulting I/Q data volume creates severe pressure on the limited interface bandwidth of low-cost SDR equipment. To ensure real-time operation, an edge-based data reduction process that forwards signal-present intervals before host transmission is essential. A fixed threshold is not suitable because detection reliability varies with the noise level. Therefore, the CA-CFAR algorithm [29], which adaptively adjusts the threshold according to the surrounding noise power, is applied in the time domain within the FPGA.

Time-domain CA-CFAR determines the detection threshold by estimating the average power of reference cells located on both sides of the cell under test (CUT). Guard cells are placed adjacent to the CUT to prevent self-masking, which occurs when the pulse edge leaks into the reference cells. With N/2 reference cells on each side of the CUT, the estimated average noise power $P_{n}$ divided by the number of total reference cells N is

(1) $$P_{n}=\left(\frac{1}{N}\right)\times \left[\sum_{i=1}^{N/2}y\left[n-G-i\right]+\sum_{i=1}^{N/2}y\left[n+G+i\right]\right]$$

where $y\left[n\right]$ denotes the instantaneous power of sample n, N is the number of reference cells, and G is the guard cell length. Assuming additive white Gaussian noise (AWGN), the target false alarm probability $P_{fa}$ is defined as the integral of the probability density function above threshold T. The threshold constant K and the final threshold T are given by

(2) $$K=N\times \left(P_{fa}^{-1/N}-1\right)$$

(3) $$T=K\times P_{n}$$

**Figure 2:**
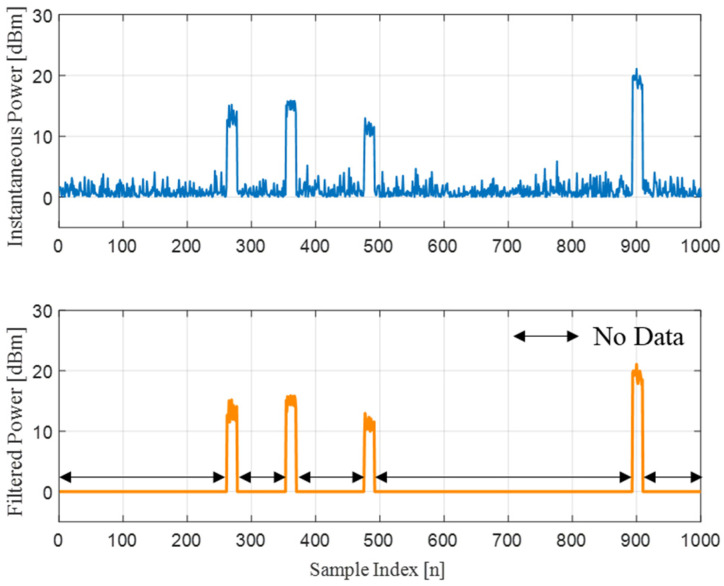
Example of the CA-CFAR algorithm in the time domain. The upper panel shows the instantaneous received power; the lower panel shows the CA-CFAR-filtered output, where noise-only intervals are discarded.

This threshold enables precise time-domain detection of pulse start and end times without prior knowledge of signal parameters. Figure 2 illustrates an example of the time-domain CA-CFAR algorithm.

In the proposed system, a CA-CFAR intellectual property (IP) core is deployed in the FPGA region of each SDR. The deployed CA-CFAR IP suppresses noise-only intervals and outputs only samples corresponding to detected pulses. Directly porting a conventional sliding-window CA-CFAR implementation onto an FPGA would result in inefficient resource usage, higher complexity and excessive memory consumption [27,28]. Conventional sliding-window CA-CFAR uses a large adder tree to sum the reference-cell data, requires a hardware-intensive divider to compute the average noise power, and consumes on-chip BRAM resources for delay line implementation. As a result, resource consumption and latency increase rapidly with window size.

To reduce this cost, the proposed CA-CFAR architecture is designed to use the limited hardware resources of the low-cost SDR FPGA as efficiently as possible. While a conventional adder tree recomputes the sum of all N elements for each window shift, yielding O(N) complexity per sample, the proposed architecture uses the following in-place recursive update rule:

(4) $$s\left[n\right]=s\left[n-1\right]+x_{new}\left[n\right]-x_{old}\left[n\right]$$

where $s\left[n\right]$ is the total power sum of the reference cells computed in the previous clock cycle, $x_{new}\left[n\right]$ is the power of the newly entered reference cell and $x_{old}\left[n\right]$ is the power of the oldest departing reference cell. This reduces the per-sample computational complexity of the reference-cell summation update to O(1) with respect to the number of reference cells, since the update uses only the newly entering and departing samples rather than recomputing the full reference window.

To compute the average noise power without a hardware divider, the number of reference cells is fixed to $2^{M}$, allowing the division operation to be replaced by

(5) $$P_{n}=\frac{1}{2^{M}}\times \sum_{i=1}^{2^{M}}P_{i}$$

with a simple arithmetic right-shift operation:

(6) $$P_{n}=\sum_{i=1}^{2^{M}}P_{i}\gg M$$

where $2^{M}$ is the total number of reference cells and $P_{i}$ is the power of the i-th reference cell within the window. When a received sample arrives at the CUT, the sample is immediately discarded if it is classified as noise. Only samples with an active detection flag are forwarded to the host PC, reducing the total transferred data volume according to the target duty cycle and thereby alleviating the transfer load on conventional low-cost SDR interfaces. Specifically, when the CA-CFAR threshold comparison classifies the CUT as a noise-only sample, the detection flag register is explicitly set to the inactive state; when the sample is subsequently passed to the output stage, the register state is checked, and inactive interval samples are discarded immediately without transmission.

**Figure 3:**
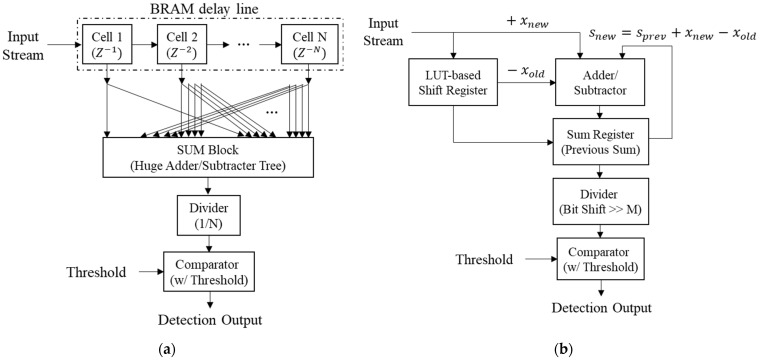
Comparison of CA-CFAR algorithm architectures: (**a**) conventional CA-CFAR using an adder tree, divider and BRAM delay line; (**b**) proposed CA-CFAR using in-place recursive summation, bit shift division and LUT-based shift registers.

In conventional FPGA-based signal processors, BRAM is primarily used to implement the sliding-window delay line; however, BRAM access introduces memory read/write latency. To avoid this latency penalty, the proposed CA-CFAR uses no BRAM. The sliding-window register chain is implemented with lookup table (LUT)-based shift registers, which are the fundamental logic elements of the FPGA. In the proposed implementation, the CA-CFAR window delay path is mapped to LUT/FF resources rather than BRAM-based storage. This resource mapping increases LUT/FF usage compared with a BRAM-based delay line while eliminating BRAM usage, reducing DSP usage, and keeping the pipeline latency fixed. On low-cost FPGAs such as the Zynq-7010, BRAM and DSP blocks are limited specialized resources shared with filters, interfaces and other processing tasks in the system. This trade-off allocates LUT/FF resources to the delay-line implementation while preserving BRAM and DSP blocks for other SDR functions. Figure 3 compares the baseline sliding-window structure and proposed hardware-efficient CA-CFAR architectures.

### 2.2. Host and GPU-Based Integrated Signal Processing Architecture

**Figure 4:**
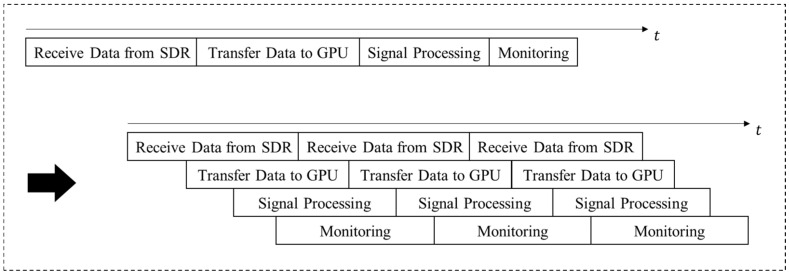
Pipelined multithreaded architecture. Each SDR node is assigned dedicated receive, transfer, signal processing and monitoring threads operating in parallel.

In an RF detection system that uses multiple distributed SDR nodes, the host must provide a management framework that can collect large volumes of data from each node without delay and reliably control all devices. For this purpose, the host software in the proposed system is designed as a multithreaded architecture. As shown in Figure 4, functions such as SDR device control, I/Q data reception and data processing are each assigned to independent threads and executed in parallel. This architecture prevents temporary delays at individual nodes from interrupting overall system operation. To minimize data loss, the dedicated reception thread is assigned the highest OS-level priority, which helps maintain stable streaming in multi-node environments.

Given that raw I/Q data flowing from the SDR to the host is a simple sample stream lacking inherent metadata, parallel processing by multiple threads makes it difficult to preserve source identity and sample ordering. To solve this data management problem, the proposed system introduces a PDU-based communication and scheduling structure. A PDU consists of a header and a payload. The header contains metadata such as center frequency, bandwidth, SDR identifier and timestamp. The payload stores valid I/Q samples passing the FPGA CA-CFAR threshold, grouped into FFT-length chunks and immediately encapsulated by the reception thread. A data storage thread communicates directly with the reception thread to save data in binary, MAT, or CSV format, while metadata is stored in separate JSONL files. For display purposes, PDUs produced by the reception thread are loaded into a priority-queue-based memory buffer, and the scheduler sorts them by metadata before forwarding them to the signal processing thread.

**Figure 5:**
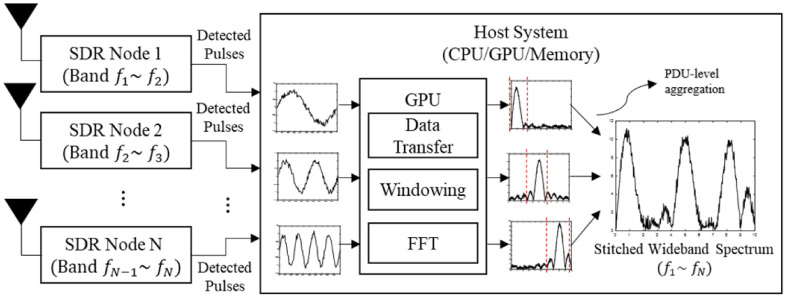
Conceptual diagram of spectrum stitching. Multiple SDR nodes each covering a sub-band (Band $f_{1}\sim f_{2},f_{2}\sim f_{3},\ldots$) and their detected events are frequency-aligned into a stitched wideband spectrum at the host GPU using PDU metadata.

Using this data management framework, the proposed system performs spectrum stitching to overcome the physical bandwidth limitation of a single low-cost SDR system [30]. As shown in Figure 5, PDUs from nodes assigned to adjacent frequency bands are sorted by metadata and aggregated into a wideband virtual spectrum map. In electronic warfare and signal collection environments in which unknown signals must be detected without prior knowledge of the frequency band or modulation scheme, this approach extends the monitoring bandwidth beyond the instantaneous bandwidth of a single low-cost SDR by aggregating spectrum observations from multiple SDR nodes. The number of SDR nodes is determined by the target monitoring bandwidth and the per-node instantaneous bandwidth.

To perform spectrum stitching and signal processing efficiently, the PDUs sorted by header are subjected to computationally intensive operations such as high-resolution FFT. If a CPU alone performs this task for data from multiple nodes, the resulting computational load limits real-time operation [25]. Therefore, the signal processing thread offloads the input PDUs to the GPU. The GPU performs windowing, FFT, magnitude computation and spectrum stitching through parallel real-time processing. The resulting spectrum is then rendered directly in the GPU’s memory.

## 3. System Implementation

**Figure 6:**
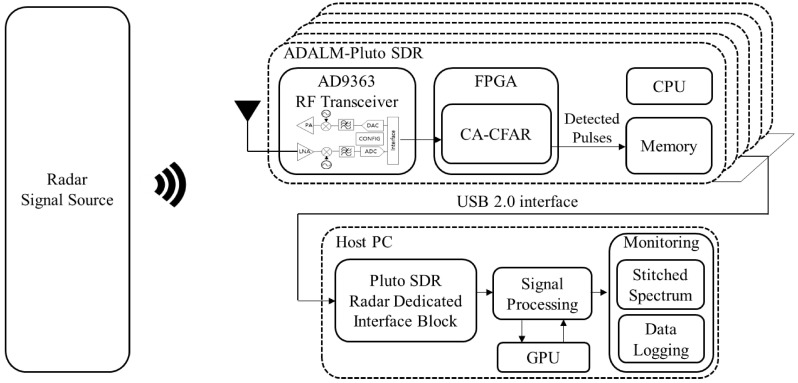
Block diagram of the proposed system hardware and software implementation. Each ADALM-Pluto SDR node contains an AD9363 RF transceiver and an FPGA integrated with the proposed CA-CFAR IP. Each node connects to the host PC via USB 2.0.

The proposed system was implemented using low-cost ADALM-Pluto SDR (Analog Devices, Inc., Wilmington, MA, USA) units [16,17] as shown in Figure 6. This section details the practical realization of the architecture presented in Section 2 across both the hardware and software layers including the FPGA CA-CFAR IP, multithreaded host operation, PDU scheduling and GPU processing pipeline.

### 3.1. CA-CFAR Implementation

Table 2 summarizes the three CA-CFAR parameter sets evaluated at the target $P_{fa}=10^{-4}$. In this table, Ref. Cell denotes the total number of reference cells, whereas Guard Cell denotes the number of guard cells on each side of the CUT. For each set, the threshold factor was calculated using Equation (2), where N is the total number of reference cells. These parameters were selected based on the actual RF operating environment. The number of reference cells used to estimate the noise power around the target signal was fixed at a power of two (64 = $2^{6}$) to enable arithmetic right-shift computation. The guard cell length was set to 12 to reduce leakage of target signal energy into adjacent reference cells.

**Figure 7:**
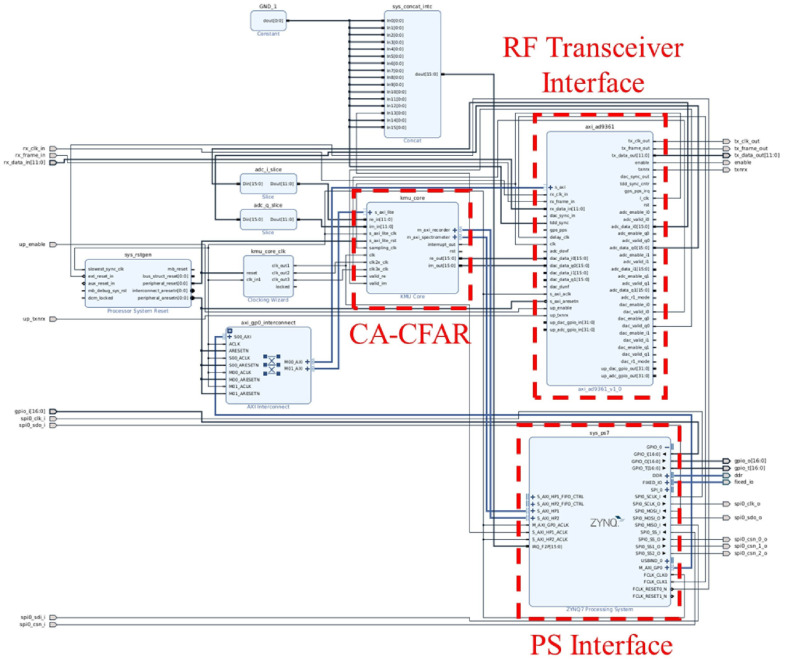
IP block diagram of the ADALM-Pluto SDR FPGA region with the proposed CA-CFAR IP inserted into the receive data path between the AD9363 transceiver and the USB output interface.

To validate the hardware-efficient CA-CFAR implementation, the CA-CFAR IP was integrated into the FPGA region of the Zynq-7010 SoC embedded in the ADALM-Pluto SDR. Development was performed using Xilinx Vitis High-Level Synthesis (HLS) and Vivado [31]. The CA-CFAR algorithm was synthesized into register-transfer-level (RTL) code and packaged as a single IP core, which was then inserted into the receive data path of the ADALM-Pluto SDR FPGA. Figure 7 shows the IP block diagram of the ADALM-Pluto SDR FPGA region with the proposed CA-CFAR IP inserted.

Table 3 compares the hardware resource utilization of the hardware-efficient CA-CFAR IP, synthesized with Vitis HLS, against a baseline sliding-window CA-CFAR implementation and the available resources of the Zynq-7010 SoC-based ADALM-Pluto SDR. As shown in Table 3, the implemented IP is suitable for low-cost FPGA environments such as the ADALM-Pluto SDR (Zynq-7010), where firmware for basic USB communication, filters and interfaces already occupies more than half of the available resources. The baseline CA-CFAR structure consumes 8 DSP blocks and 2 BRAM blocks and incurs 487 clock cycles. By contrast, the proposed architecture uses no BRAM and only 4 DSP blocks—the scarcest on-chip resources—and occupies 16.3% of the available LUTs and 11.6% of the available FFs. Compared with the baseline implementation, the proposed design reduces the latency from 487 to 10 clock cycles at 100 MHz, corresponding to an approximately 49-fold reduction. The higher LUT/FF utilization mainly results from the LUT-based shift-register delay path and additional pipeline/control registers used for detection-flag propagation and edge-side sample suppression. As a result, LUT usage increases from 1825 to 2861 and FF usage from 967 to 4068 compared with the baseline implementation, while BRAM usage is eliminated, DSP usage is reduced from 8 to 4 blocks, and latency is reduced from 487 to 10 clock cycles at 100 MHz.

### 3.2. Multithreaded Operation and GPU Signal Processing Implementation

The multithreaded operation scheme is implemented in C/C++. The signal processing thread uses the CUDA API to exchange data with the GPU. Pinned memory and asynchronous transfers are employed to minimize latency caused by CPU-GPU memory copies. Pinned memory refers to a fixed memory region that is not subject to OS page replacement; by keeping the physical address of the data fixed during transfer, it allows the DMA controller to access memory directly without intermediate buffering. In contrast, conventional pageable memory requires the OS to copy data to an internal staging buffer before initiating DMA transfer; pinned allocation eliminates this intermediate copy step entirely, reducing both transfer latency and CPU involvement. Asynchronous transfer allows the CPU to proceed immediately to the next task after issuing a GPU transfer command, without blocking until the transfer completes. Together, these techniques reduce memory-copy overhead and support pipelined RF monitoring.

The GPU performs windowing and FFT on input data arranged in PDU-length units sorted by the PDU header. The maximum sampling frequency of the ADALM-Pluto SDR is 61.44 MSps, yielding a frequency resolution of

(7) $$∆f=\frac{f_{s}}{N}=\frac{61.44\;MHz}{1024}=60\;kHz$$

where $f_{s}$ is the sampling frequency and N is the FFT size. After the FFT, spectrum stitching is performed in the frequency domain. Each SDR is assigned to a non-overlapping sub-band, and the host aggregates the detected events using PDU metadata, including the SDR identifier, center frequency and timestamp to preserve source identity, order PDU chunks and place detected events on the stitched frequency axis. Within each node, the time axis resolution $T_{res}$ is determined by the reciprocal of the sampling rate:

(8) $$T_{res}=\frac{1}{f_{s}}=\frac{1}{61.44\;MHz}\approx 16.276\;ns$$

The resulting sample interval is 16.276 ns at 61.44 MSps. This value represents the temporal granularity for localizing events within each node’s PDU stream. Equations (7) and (8) together define the frequency-bin spacing and per-node sample interval used by the PDU-level wideband monitoring pipeline.

## 4. Experimental Results and Discussion

### 4.1. Testbed and Experimental Setup

**Figure 8:**
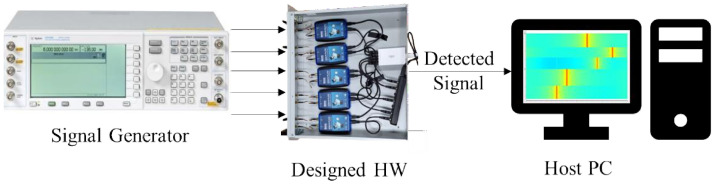
Block diagram of the experimental setup, showing signal flow and system components.

Figure 8 shows the block diagram of the experimental setup. The hardware-efficient CA-CFAR IP deployed in the FPGA was first verified to accurately identify and output only valid RF signal intervals while suppressing noise-only intervals. Experiments were conducted by transmitting linear frequency-modulated (LFM) pulse signals from a signal generator and receiving them with a self-built hardware testbed using five ADALM-Pluto SDR units.

### 4.2. Wideband Coverage and Data Reduction

**Figure 9:**
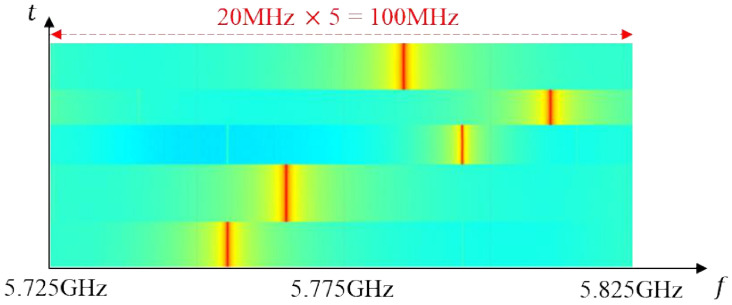
Real-time wideband spectrogram of the proposed system covering 100 MHz (5.725–5.825 GHz) using five ADALM-Pluto SDR nodes. Only detected pulses are displayed; noise-only intervals are discarded by the edge CA-CFAR IP.

Figure 9 shows that events with different center frequencies, bandwidths, pulse widths, and received power levels are detected and placed in their corresponding sub-bands within the 100 MHz stitched view. The five ADALM-Pluto SDR nodes were configured with center frequencies spanning the C-band from 5.725 GHz to 5.825 GHz—a band predominantly occupied by drone control and video transmission signals. Each node covered 20 MHz, providing a combined coverage of 100 MHz. To evaluate signal-selective forwarding by the proposed system, five pulses with arbitrary pulse widths, bandwidths, center frequencies and power levels sufficiently above the detection threshold were transmitted.

The experimental results show that, after passing through the CA-CFAR IP, signal output was activated only during pulse intervals whose energy exceeded the detection threshold, while samples from noise-only intervals were suppressed at the SDR stage. For each detected event, pulse descriptor words (PDWs)—including arrival time and pulse width—are accessible through the internal PDU metadata.

To characterize the duty-cycle dependence of the SDR-side data reduction, the signal duty cycle was set to 5%, 10%, 20%, 30%, and 50%. The sampling rate, pulse repetition interval (PRI), received signal power, CA-CFAR configuration, and recording duration were kept constant across all conditions. The sampling rate was set to 20 MSps; the PRI was 100 μs; and the corresponding pulse widths were 5, 10, 20, 30, and 50 μs. Each recording was performed for one second using the FPGA-implemented Set B configuration. For each duty-cycle condition, the edge-reduced data volume received at the host was recorded. At 20 MSps with 16-bit I and 16-bit Q samples, the calculated raw-transfer rate is 80.00 MB/s. The edge-transfer rate was calculated from the data volume received at the host and the acquisition duration, and the data-reduction ratio was also calculated relative to the 80.00 MB/s raw-transfer baseline.

**Figure 10:**
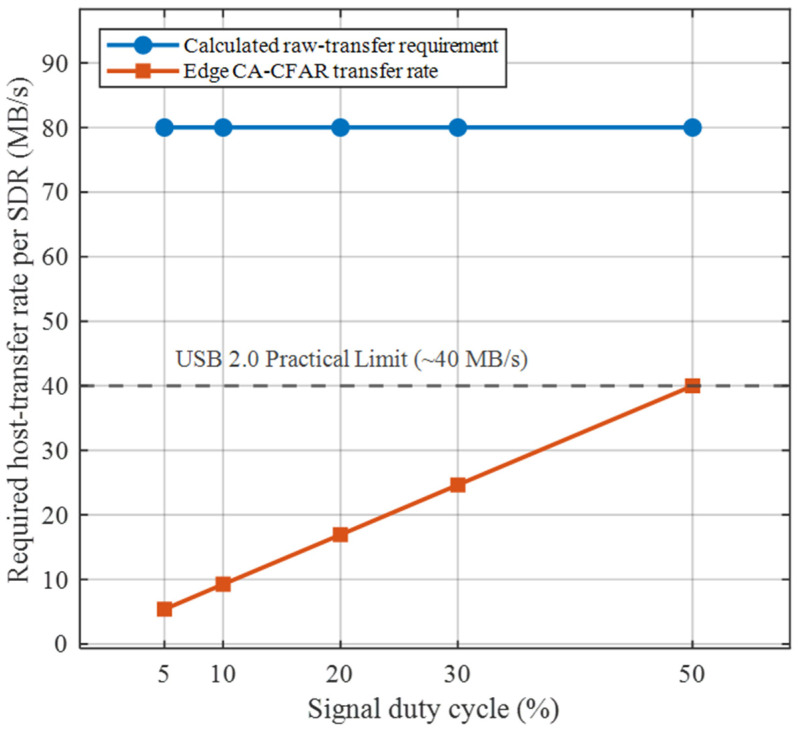
Calculated raw-transfer requirement and measured edge CA-CFAR transfer rate per SDR as a function of signal duty cycle. The raw-transfer requirement is fixed by the 20 MSps sampling configuration, whereas the measured edge-transfer rate increases with the signal-present interval. The dashed horizontal line indicates the practical USB 2.0 reference limit of approximately 40 MB/s. All conditions use the FPGA-implemented Set B configuration.

As shown in Figure 10, the required edge-transfer rates were 5.39, 9.26, 16.93, 24.65, and 39.99 MB/s at duty cycles of 5%, 10%, 20%, 30%, and 50%, respectively. These values correspond to data-reduction ratios of 93.27%, 88.43%, 78.84%, 69.19%, and 50.02%, respectively. As the signal occupancy increased, longer signal-present intervals had to be retained and transferred to the host, resulting in a higher edge-transfer rate and a lower data-reduction ratio. The edge-transfer rate remained below the practical USB 2.0 limit at duty cycles up to 30% and approached the approximately 40 MB/s limit at a 50% duty cycle. In contrast, the calculated raw-transfer requirement of 80.00 MB/s exceeded this practical limit under all evaluated conditions. Each condition was repeated three times, and the reported values represent the means of the repeated measurements.

### 4.3. CA-CFAR Detection Performance

**Figure 11:**
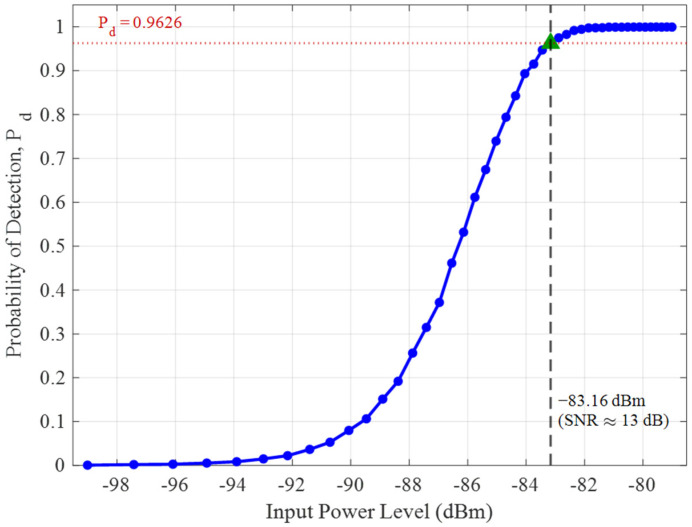
Measured probability of detection $\left(P_{d}\right)$ versus input power level. At a noise floor of −96 dBm, $P_{d}$ first exceeds 95% ($P_{d}$ = 96.26%) at −83.16 dBm, indicating a minimum detectable SNR of approximately 13 dB.

CA-CFAR detection performance was characterized by measuring detection probability as a function of received signal power. The experiment was conducted at room temperature using a signal generator connected via an SMA coaxial cable. The input signal was configured as a pulse train with a pulse repetition interval (PRI) of 100 μs and a duty cycle of 10% (pulse width of 10 μs), and detection performance was measured while the signal power was varied. The FFT size was set to 1024 for signal processing, and the average noise floor was measured to be −96 dBm. As shown in Figure 11, the detection probability increases gradually with received power, reaching approximately 50% at −86.1 dBm. For signal power above −83.16 dBm, the detection probability first exceeds 95%, achieving $P_{d}$ = 96.26%, and approaches 99% above −82.4 dBm. Under the tested condition, the minimum detectable SNR was approximately 13 dB.

The detection–false alarm trade-off of the proposed CA-CFAR detector was characterized over the evaluated threshold range using receiver operating characteristic (ROC) curves generated by Monte Carlo simulation. The simulation replicates the exact fixed-point integer pipeline of the FPGA IP core: 16-bit signed I/Q samples, power metric $I^{2}+Q^{2}$ implementation using four DSP blocks, accumulation over $N_{total}\;$= 64 reference cells with exact division by a 6-bit right-shift and Q8 threshold multiplication ($K_{num}=2536,\;\gg 8$). Each ROC curve is traced by sweeping the threshold factor K over a logarithmic grid and recording the empirical $P_{fa}$ and $P_{d}$ from $M=10^{8}$ independent trials per operating point, ensuring a statistical resolution of approximately $1\times 10^{-8}$ in $P_{fa}$.

The threshold factor K = 9.9063 implemented in the hardware was derived from the standard CA-CFAR false alarm formula $P_{fa}={\left(1+K/N\right)}^{-N}$ with N=64 and $P_{fa}\approx 10^{-4}$. While the theoretical formula assumes continuous precision, the hardware-exact simulation identifies that $K_{fp}=\;9.9063$ yields an empirical $P_{fa}$ of $1.24\times 10^{-4}$. A slightly higher calibrated constant, $K_{emp}=10.15$, is found to achieve $P_{fa}=10^{-4}$ precisely. The small deviation in the empirical $P_{fa}$ ($1.24\times 10^{-4}\;vs.\;10^{-4})$ arises from fixed-point quantization of the Q8 multiplier and is well within the acceptable false alarm tolerance of the system.

**Figure 12:**
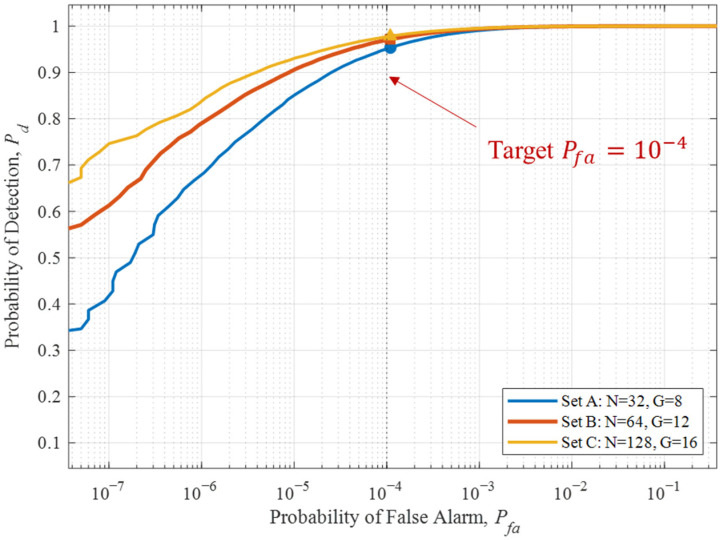
Fixed-point Monte Carlo CA-CFAR results using $10^{8}$ trials per point and target $P_{fa}=10^{-4}$; parameter set ROC curves at SNR ≈ 13 dB (−83.16 dBm; noise floor −96 dBm) for Set A, B, and C. The markers indicate each set’s Q8 design threshold.

Figure 12 compares the three parameter sets at SNR = 13 dB. At their respective Q8 design thresholds, Sets A, B, and C achieve $P_{d}$ values of 0.9531, 0.9715, and 0.9781, respectively, while their empirical $P_{fa}$ values remain close to the target of $10^{-4}$.

The ROC curves in Figure 12 also explain the transition observed near the minimum detectable SNR. At the design threshold, increasing the SNR from 11 dB to 13 dB raises $P_{d}$ from approximately 0.75 to 0.97. This transition is consistent with the measured detection onset above −83.16 dBm in Figure 11. Among these configurations, Set B was used for the FPGA implementation and subsequent RF experiments.

### 4.4. GPU Acceleration and Real-Time Latency

After validating the FPGA CA-CFAR stage, we evaluated the processing latency of the proposed GPU-accelerated processing pipeline and the system-level latency from signal acquisition to display-buffer preparation. Since the proposed multi-SDR-based distributed RF surveillance system must process wideband spectrum data collected from multiple nodes in real time, minimizing the system-level latency is an important system metric. The computational latency of CPU-only processing was compared with that of the proposed GPU-accelerated pipeline. Memory copies between the CPU and GPU were performed using pinned memory and asynchronous transfers.

**Figure 13:**
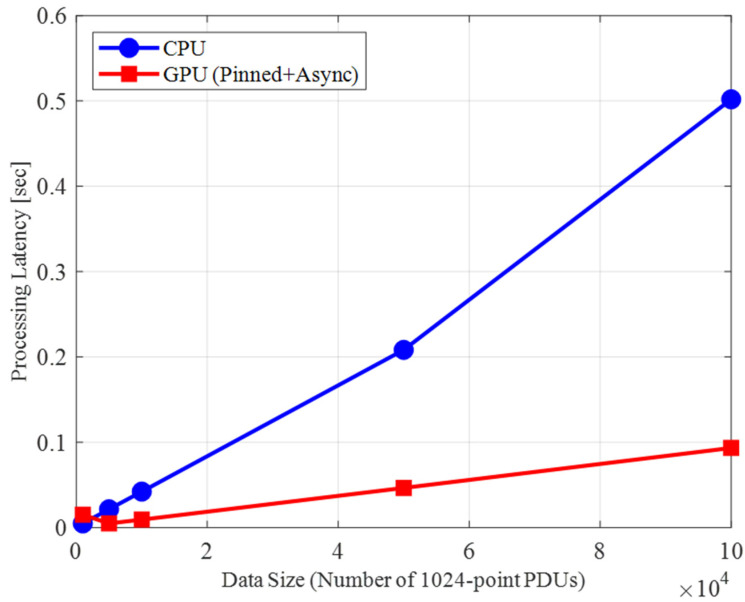
CPU and GPU (pinned + asynchronous transfer) processing times as a function of the number of 1024-point PDUs. At larger PDU counts, the tested GPU pipeline achieved a 4.5× to 6.0× processing-time reduction relative to the tested CPU implementation.

Figure 13 shows processing latency as a function of the number of 1024-point PDUs, representing the cumulative received data volume from multiple nodes. The comparison is specific to the tested implementations, workload, and hardware configuration, and the host PC was equipped with an Intel i7-12700, 16 GB of RAM and an NVIDIA GeForce RTX 3050 GPU. Performance evaluation was conducted on the actual hardware pipeline, including windowing, 1024-point FFT, magnitude computation and display-buffer preparation. GPU pre-warmup was performed before timing to exclude first-run overhead.

For the smallest evaluated workload of 1000 PDUs, GPU execution was dominated by host–device memory-transfer overhead. At 100,000 PDUs, however, the GPU-accelerated pipeline achieved a 4.5× to 6.0× latency reduction because the larger workload amortized this transfer overhead. The performance gain at large PDU counts comes mainly from reducing data movement and keeping windowing, FFT, magnitude computation, and display preparation in GPU memory.

Real-time processing is important for RF signal detection systems, and system-level latency is a key performance indicator. In this work, latency was characterized as the steady-state time required to process a single 1024-sample PDU from SDR sample acquisition to GPU-side display-buffer preparation. Latency analysis was performed by separating hardware-imposed constraints from software computation time. The experiment was conducted in 1024-sample units with both PDU size and FFT size set to 1024. At a sampling rate of 20 MSps, the calculated buffer-filling time was 51.2 μs. The USB transfer contribution was set to 125 μs based on the high-speed micro-frame cadence observed in the device-to-host transfer log.

The measurement assumed that only the proposed system was running on the host PC, reducing the influence of nondeterministic OS scheduling overhead. The receiver and GPU were initialized before measurement, and the GPU pipeline was warmed up to complete CUDA initialization, FFT setup, and memory allocation. Five measurement runs were performed, with 1000 iterations per run. The GPU signal processing and display-buffer preparation components were profiled separately using CUDA timing events. The timed pipeline included host-to-device transfer, windowing, FFT, magnitude calculation, display-buffer preparation, and CUDA stream synchronization. The reported value represents steady-state processing after receiver initialization. The calculated buffer-filling time and the fixed USB micro-frame interval were added separately to the measured GPU pipeline time. The result is summarized in Table 4.

Across the 5000 measurements, the latency from signal acquisition to display-buffer preparation had a mean of 0.376 ms and a standard deviation of 0.060 ms. The median and 95th-percentile latencies were 0.355 and 0.533 ms, respectively, while the 99th percentile was 0.607 ms. The maximum observed latency was 0.822 ms, and all measured values remained below 1 ms under the tested latency model. The median latency of the five runs ranged from 0.351 to 0.362 ms. The coefficient of variation among the run-level medians was 1.573%, showing limited variation between repeated runs.

## 5. Conclusions

This paper proposed a bandwidth-efficient real-time wideband RF spectrum sensing system based on multiple low-cost SDRs and FPGA-GPU heterogeneous acceleration, with performance experimentally verified on a custom hardware testbed. The two main contributions of this work can be summarized as follows.

First, a hardware-efficient CA-CFAR IP was implemented on the FPGA of low-cost SDRs in order to perform edge-side selective data reduction, achieving 88% reduction in transmitted data volume in a 10% duty-cycle environment and reducing host-interface bandwidth demand.

Second, five tuned low-cost SDRs were operated in parallel, and their detected events were combined using PDU metadata including SDR identifier, center frequency, bandwidth, and timestamp, achieving 100 MHz wideband detection coverage in the 5.8 GHz C-band.

In the system-level evaluation, the PDU-based GPU pipeline achieved a 4.5× to 6.0× processing-time reduction relative to the tested CPU baseline and a latency of 376 μs from signal acquisition to display-buffer preparation.

In the CA-CFAR detection performance evaluation, the measured probability of detection exceeded 95% at −83.16 dBm received power, with $P_{d}$ = 96.26%, corresponding to a minimum detectable SNR of approximately 13 dB. These results indicate that the proposed architecture can support real-time wideband RF monitoring under the evaluated low-cost multi-SDR configuration. Future work will focus on integrating RF signal processing with an FPGA-based deep learning inference engine on the RFSoC platform to extend the system into an intelligent real-time signal processor with pulse parameter estimation and signal classification capabilities.

## Figures and Tables

**Table 1:** Category-level qualitative comparison of related spectrum-monitoring and signal-processing approaches.

Category	Example Refs.	Platform	Processing Location	Host-Interface Data Flow	Wideband Support
SDR monitoring	[13,14,15,16,17]	SDR + host	Host PC	Study dependent	Limited by node bandwidth and interface
GPU processing	[18,19,20,22,23,24,25]	Host CPU/GPU	Host GPU	Data transferred before GPU processing	Study dependent
FPGA-assisted detection	[21,26]	FPGA/front end	Front end or FPGA	Application dependent	Application dependent
This work	This work	Low-cost SDR FPGA + host GPU	SDR FPGA + host GPU	CA-CFAR-selected intervals transferred	100 MHz aggregate view using five 20 MHz nodes

**Table 2:** CA-CFAR parameter sets evaluated at the target $P_{fa}=10^{-4}$.

Set	Ref. Cell	Guard Cell	K
A	32	8	10.6727
B	64	12	9.9063
C	128	16	9.5498

**Table 3:** Hardware resource utilization of the proposed CA-CFAR IP vs. total resources of the ADALM-Pluto SDR (Zynq-7010).

	Latency (Clocks)	BRAM	DSP	FF	LUT
Pluto SDR (Total)	-	60	80	35,200	17,600
Conventional CA-CFAR	487	2	8	967	1825
Proposed CA-CFAR	10	0	4	4068	2861

**Table 4:** Steady-state latency from signal acquisition to display-buffer preparation for a single 1024-sample PDU.

Stage	Component	Duration (ms)
Data acquisition	Analog buffer filling	0.051
Data transfer	USB micro-frame transfer interval	0.125
GPU data transfer	Host-to-device transfer	0.071
Signal processing	GPU signal processing	0.080
Display preparation	Display-buffer preparation	0.026
Software overhead	CUDA dispatch and synchronization	0.022
Total	Total steady-state latency	0.376

## Data Availability

The source code supporting the results reported in this study is openly available on GitHub at https://github.com/baeyoonsoo/spectrum_sensing_system (accessed on 10 July 2026), including the FPGA CA-CFAR IP core (Vitis HLS), GPU-accelerated processing pipeline, Monte Carlo ROC simulation, and latency measurement script. Note that duration_check.py measures GPU signal processing and display-buffer preparation as a combined stage and uses a GPU buffer copy to approximate display-buffer preparation for hardware-independent execution; the individual Table 4 values were profiled using CUDA timing events in the full display-connected system. The processed $P_{d}$-versus-input-power data used to generate Figure 11 (pd_vs_power_fig11.csv) were obtained from the conducted RF measurements described in Section 4.3 and included in the GitHub repository. The CPU vs. GPU latency benchmark (CPU_vs_GPU_computing_time.m, Figure 13) uses randomly generated input data without a fixed seed; absolute timing values therefore vary across runs due to data randomness and OS scheduling, but the GPU speedup over CPU consistently falls in the 4.5× to 6.0× range at large PDU counts. Raw RF measurement data from the physical hardware testbed are not publicly available as the hardware testbed noise environment cannot be exactly reproduced; these data are available from the corresponding author upon reasonable request.

## References

[1] Yucek T., Arslan H. (2009). A survey of spectrum sensing algorithms for cognitive radio applications. IEEE Commun. Surv. Tutor..

[2] Riviello D.G., Alfano G. (2026). Eigenbased Multi-Antenna Spectrum Sensing: Experimental Validation on a Software-Defined Radio Testbed. Sensors.

[3] Axell E., Leus G., Larsson E.G., Poor H.V. (2012). Spectrum sensing for cognitive radio: State-of-the-art and recent advances. IEEE Signal Process. Mag..

[4] Xiao Q. A conceptual architecture of cognitive electronic warfare system. Proceedings of the 10th International Conference on Advanced Cognitive Technologies and Applications (COGNITIVE).

[5] Guvenc I., Koohifar F., Singh S., Sichitiu M.L., Matolak D. (2018). Detection, tracking and interdiction for amateur drones. IEEE Commun. Mag..

[6] Liou L.L., Lin D.M., Tsui J.B., Hary S. Wideband signal detection by employing differential sampling rates. Proceedings of the 2011 IEEE National Aerospace and Electronics Conference (NAECON).

[7] Alam S.S., Chakma A., Rahman M.H., Bin Mofidul R., Alam M.M., Utama I.B.K.Y., Jang Y.M. (2023). RF-enabled deep-learning-assisted drone detection and identification: An end-to-end approach. Sensors.

[8] Elyousseph H., Altamimi M. (2024). Robustness of Deep-Learning-Based RF UAV Detectors. Sensors.

[9] Molina-Tenorio Y., Prieto-Guerrero A., Aguilar-Gonzalez R. (2021). Real-time implementation of multiband spectrum sensing using SDR technology. Sensors.

[10] Nasser A., Al Haj Hassan H., Abou Chaaya J., Mansour A., Yao K.-C. (2021). Spectrum sensing for cognitive radio: Recent advances and future challenge. Sensors.

[11] Jeon J., Seo B.-S., Ju Y., Lim K.-C., Lee S.-J. (2023). Development of a real-time spectrum analyzer for radar pulse signals using Xilinx RFSoC. J. Korean Inst. Electromagn. Eng. Sci..

[12] Cha M., Choi H., Kim S., Moon B., Kim J., Lee J. (2019). Development of a digital receiver for detecting radar signals. J. Korea Inst. Mil. Sci. Technol..

[13] Chiper F.-L., Martian A., Vladeanu C., Marghescu I., Craciunescu R., Fratu O. (2022). Drone detection and defense systems: Survey and a software-defined radio-based solution. Sensors.

[14] Ferreira L.S., Santos J.F.C.M., Carvalho N.B. (2026). Wideband monitoring system of drone emissions based on SDR technology with RFNoC architecture. Drones.

[15] Sharma S.K., Bogale T.E., Chatzinotas S., Ottersten B., Le L.B., Wang X. (2015). Cognitive radio techniques under practical imperfections: A survey. IEEE Commun. Surv. Tutor..

[16] Han S., Park H., Bae Y., Lee H., Cho Y.-K., Choi W., Kim H.-R., Jang B.-J. (2026). Application of low-cost Pluto SDR as a radar target simulator. J. Korean Inst. Electromagn. Eng. Sci..

[17] Wu Z., Qaragoez Y., Volskiy V., Huangfu J., Ran L., Schreurs D. (2022). A joint design of radar sensing, wireless power transfer, and communication based on reconfigurable software defined radio. Electronics.

[18] Liu G., Yue N., Wang S. (2023). A GPU-based real-time processing system for frequency division multiple-input-multiple-output radar. IET Radar Sonar Navig..

[19] Zhao X., Liu P., Wang B., Jin Y. (2023). GPU-accelerated signal processing for passive bistatic radar. Remote Sens..

[20] Rupniewski M., Mazurek G., Gambrych J., Nalecz M., Karolewski R. A real-time embedded heterogeneous GPU/FPGA parallel system for radar signal processing. Proceedings of the 2016 IEEE International Conferences on Ubiquitous Intelligence and Computing (UIC/ATC/ScalCom).

[21] Sim Y., Heo J., Jung Y., Lee S., Jung Y. (2023). FPGA implementation of efficient CFAR algorithm for radar systems. Sensors.

[22] Venter C.J., Grobler H., AlMalki K.A. Implementation of the CA-CFAR algorithm for pulsed-Doppler radar on a GPU architecture. Proceedings of the 2011 IEEE Jordan Conference on Applied Electrical Engineering and Computing Technologies (AEECT).

[23] Andrews G.S., Shake T.H. Real-time, GPU-accelerated processing of digital radar signal data. Proceedings of the 2012 IEEE Radar Conference.

[24] Dong H., Zheng C., Tian W. (2020). Research on parallel architecture design of radar real-time signal processing based on CPU-GPU heterogeneous platform. IET Conference Proceedings CP779.

[25] Perdana R.S., Sitohang B., Suksmono A.B. A survey of graphics processing unit (GPU) utilization for radar signal and data processing system. Proceedings of the 2017 6th International Conference on Electrical Engineering and Informatics (ICEEI).

[26] Wang Y., Liu Q., Fathy A.E. (2013). CW and pulse-Doppler radar processing based on FPGA for human sensing applications. IEEE Trans. Geosci. Remote Sens..

[27] Richards M.A. (2014). Fundamentals of Radar Signal Processing.

[28] Urkowitz H. (1967). Energy detection of unknown deterministic signals. Proc. IEEE.

[29] Rohling H. (1983). Radar CFAR thresholding in clutter and multiple target situations. IEEE Trans. Aerosp. Electron. Syst..

[30] Uvaydov D., Zhang M., Robinson C.P., D’Oro S., Melodia T., Restuccia F. Stitching the Spectrum: Semantic Spectrum Segmentation with Wideband Signal Stitching. Proceedings of the IEEE INFOCOM 2024.

[31] Fisne A., Bahceci M.U., Dursun M., Aydogmus S., Cetintepe C. High-Level Synthesis based radar hardware implementation and performance comparison. Proceedings of the 2022 Innovations in Intelligent Systems and Applications Conference (ASYU).

